# Impact of Tumor Size on Outcomes of Hepatic Arteriography and C-Arm CT-Guided Ablation (HepACAGA): > 3 cm Is No Absolute Contraindication

**DOI:** 10.1007/s00270-025-04167-8

**Published:** 2025-08-26

**Authors:** Niek Wijnen, Emma Ruijs, Rutger C. G. Bruijnen, Joep de Bruijne, Jeroen Hagendoorn, Guus M. Bol, Martijn P. W. Intven, Maarten L. J. Smits

**Affiliations:** 1 Department of Radiology and Nuclear Medicine, University Medical Center Utrecht, 3584 CX Utrecht, The Netherlands; 2 Department of Gastroenterology and Hepatology, University Medical Center Utrecht, 3584 CX Utrecht, The Netherlands; 3 Department of Surgery, University Medical Center Utrecht, 3584 CX Utrecht, The Netherlands; 4 Department of Medical Oncology, University Medical Center Utrecht, 3584 CX Utrecht, The Netherlands; 5 Division of Hematology/Oncology, Department of Medicine, University of California, San Francisco, CA USA; 6 Department of Radiotherapy, University Medical Center Utrecht, 3584 CX Utrecht, The Netherlands

**Keywords:** C-arm CT, CTHA, Colorectal liver metastases, Hepatic arteriography, Hepatocellular carcinoma, Microwave ablation

## Abstract

**Purpose:**

A tumor diameter > 3 cm is considered a relative contraindication for thermal ablation due to a significant risk of post-ablation recurrence. However, current advanced ablation techniques might allow for successful ablation of larger tumors. This study aimed to evaluate the impact of tumor size on outcomes of Hepatic Arteriography and C-Arm CT-Guided Ablation (HepACAGA).

**Methods:**

Patients treated with HepACAGA for hepatocellular carcinoma (HCC) or colorectal liver metastases (CRLM) between January 2021 and June 2025 were analyzed. All ablations were performed with microwave ablation. Patients were stratified by tumor size: ≤ 2 cm, 2–3 cm, and 3–5 cm. Outcomes assessed included local tumor progression-free survival (LTPFS), local tumor progression (LTP) rate, and complications.

**Results:**

A total of 137 consecutive patients with 265 tumors (152 HCC and 113 CRLM) were included: 187 tumors ≤ 2 cm, 52 tumors 2–3 cm, and 26 tumors 3–5 cm. The 1-year LTPFS was most favorable for tumors ≤ 2 cm (96%; 95% CI: 93–99), followed by 2–3 cm (93%; 95% CI: 85–100), and 3–5 cm (90%; 95% CI: 78–100). No significant differences in LTPFS were found (*p* = 0.580). Overall, LTP occurred in 5% of tumors. Secondary LTP rates were 3% for tumors ≤ 2 cm and 4% for both tumors 2–3 cm and 3–5 cm (*p* = 0.966). Complication rates were 4% for tumors ≤ 2 cm, 6% for tumors 2–3 cm, and 13% for tumors 3–5 cm (*p* = 0.236).

**Conclusion:**

HepACAGA proved to be effective and safe for treating patients with HCC and CRLM across a broad range of tumor sizes. These findings suggest that intermediate-sized tumors (3–5 cm) could be eligible for thermal ablation without compromising post-ablation recurrence.

**Graphical Abstract:**

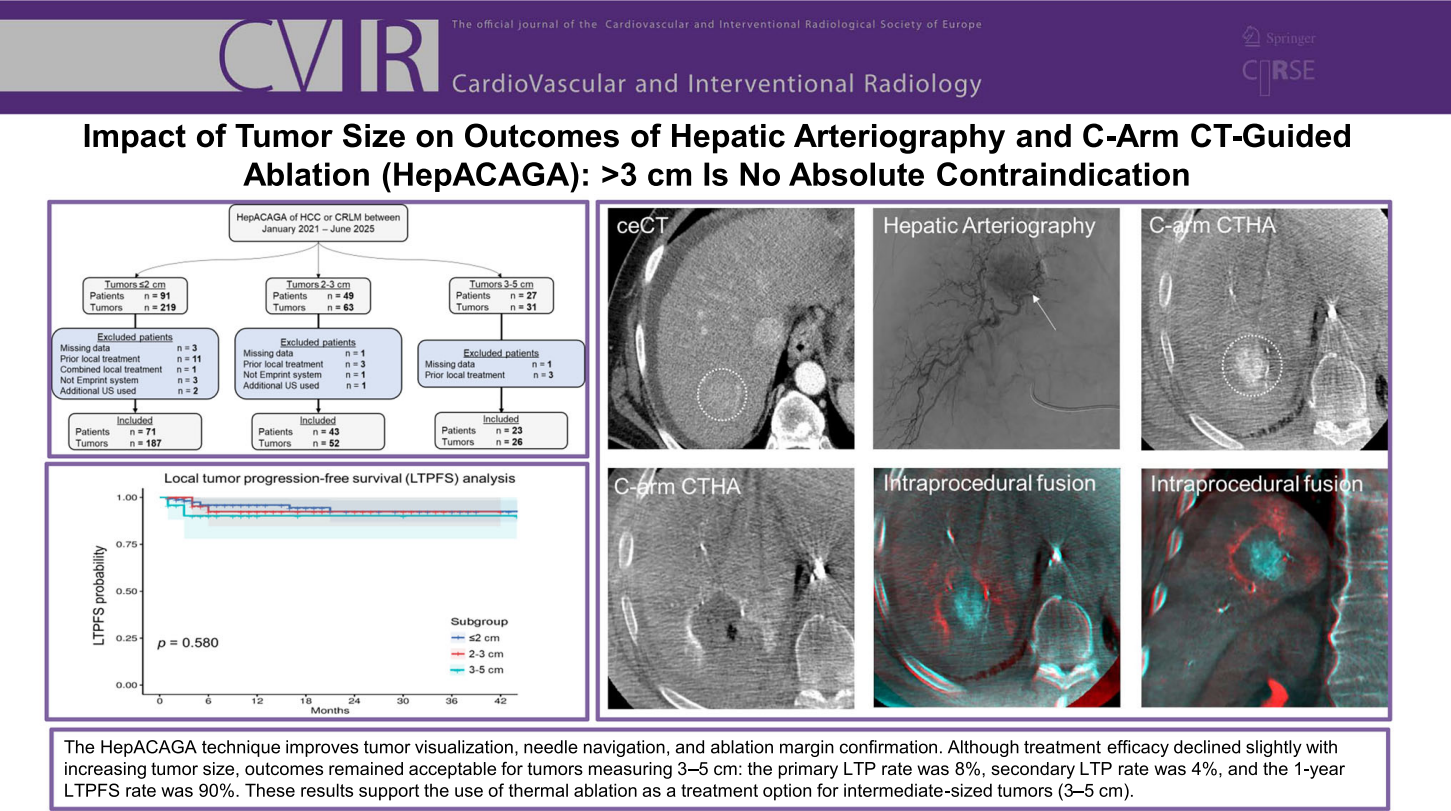

**Supplementary Information:**

The online version contains supplementary material available at 10.1007/s00270-025-04167-8.

## Introduction

Thermal ablation is an effective locoregional treatment modality for primary liver tumors and liver metastases ≤ 3 cm in diameter [1, 2]. The 3 cm-diameter threshold is primarily based on older studies using outdated ablation modalities and image-guidance technologies [3–5]. Modern image-guidance techniques enable more complex needle placement and precise ablation margin assessment [6].

Technological advancements in thermal ablation have significantly increased over the past years leading to improved ablation technologies [7]. Microwave ablation (MWA) has largely replaced radiofrequency ablation (RFA) due to advantages including more homogenous tissue heating, spherical ablation zones, and reduced susceptibility to the heat-sink effect [8, 9]. High-power MWA systems have further enhanced ablation efficacy by enabling the creation of a 5 cm (short axis) ablation zone from a single needle position [10]. This is particularly important because the 3 cm limit is largely attributed to the difficulty of generating a homogeneous area of coagulative necrosis with adequate margins for larger tumors.

In addition to advancements in ablation technologies, more accurate image-guidance, needle-placement and ablation margin assessment techniques have been developed [6, 7]. A significant advancement in intraprocedural tumor visualization and ablation zone assessment is the introduction of CT hepatic arteriography (CTHA) for ablation procedures. This technique involves inserting a catheter into the hepatic artery and performing (C-arm) CT imaging during intra-arterial contrast injection. The use of CTHA has greatly improved outcomes in liver tumor ablations [6, 11, 12]. At our center, the HepACAGA technique (Hepatic Arteriography and C-Arm CT-Guided Ablation) is employed, which combines C-arm CT hepatic arteriography (C-arm CTHA) with C-arm CT-guided microwave ablation [13–15]. In a recent comparative analysis, it was demonstrated that the enhanced tumor visualization, needle navigation, and ablation margin confirmation obtained with the HepACAGA technique significantly improved local tumor progression free survival, while reducing complication rates compared to conventional US-/CT-guided ablation [15].

Despite these significant advancements in ablation techniques, many guidelines still adhere to the 3 cm limit for thermal ablation [16–18]. This underscores the need to reassess whether lesions > 3 cm should still be considered a contraindication for thermal ablation using techniques available in current clinical practice. The aim of this study was to evaluate the impact of tumor size on outcomes of the HepACAGA technique.

## Methods

### Ethical Approval

Data were extracted from the *Minimally Invasive Thermal Ablation (MISTRAL)* study, a prospective registry of liver tumor ablations at the University Medical Center Utrecht (Utrecht, The Netherlands). This prospective study was approved by the local ethical institutional review board (no. 21/709). All included patients provided written informed consent. Some patients may overlap with previously published analyses from the MISTRAL database that investigated different study objectives [14, 15].

### Study Population

All consecutive patients who underwent HepACAGA for hepatocellular carcinoma (HCC) or colorectal liver metastases (CRLM) between January 2021 and June 2025 were included.

The following exclusion criteria were applied: (1) missing data; (2) ablations of recurrent tumors after prior local treatment; (3) ablations combined with another local treatment; (4) additional use of US; (5) MWA performed with a system other than Emprint. Patients without follow-up imaging were only included in the technical success analysis.

### HepACAGA Procedure

The entire HepACAGA procedure was performed in an angiography suite with patients under general anesthesia [13]. Arterial access was achieved via femoral or radial artery puncture, and a catheter was advanced into the common, proper, left or right hepatic artery, depending on tumor location. CTHA was obtained by performing C-arm CT during intra-arterial contrast injection. C-arm CT was performed using a monoplane C-arm system (Allura FD20 Xper, Philips, Best, the Netherlands) in propeller position with an ‘open trajectory rotation’ (240° rotation, 308 projections) and 10.4 s rotation time (XperCT HD fast setting). Contrast agent (Visipaque 320 mg/ml, 2:1 diluted with NaCl) was power-injected at 1.0–2.0 ml/s (total amount 10–40 mL) with a 10-s delay.

C-arm CTHA was checked for tumor visualization and assessment of nearby critical non-target structures. C-arm navigation software (XperGuide, Philips, Best, The Netherlands) was employed for needle trajectory planning. Next, the needle was inserted under real-time fluoroscopic guidance along the planned trajectory. C-arm CT acquisition and needle placement were performed under apnea (pausing the ventilator) in order to obtain a near-identical liver position. C-arm CTHA was repeated to confirm accurate needle placement, with needle repositioning if necessary.

**Fig. 1:**
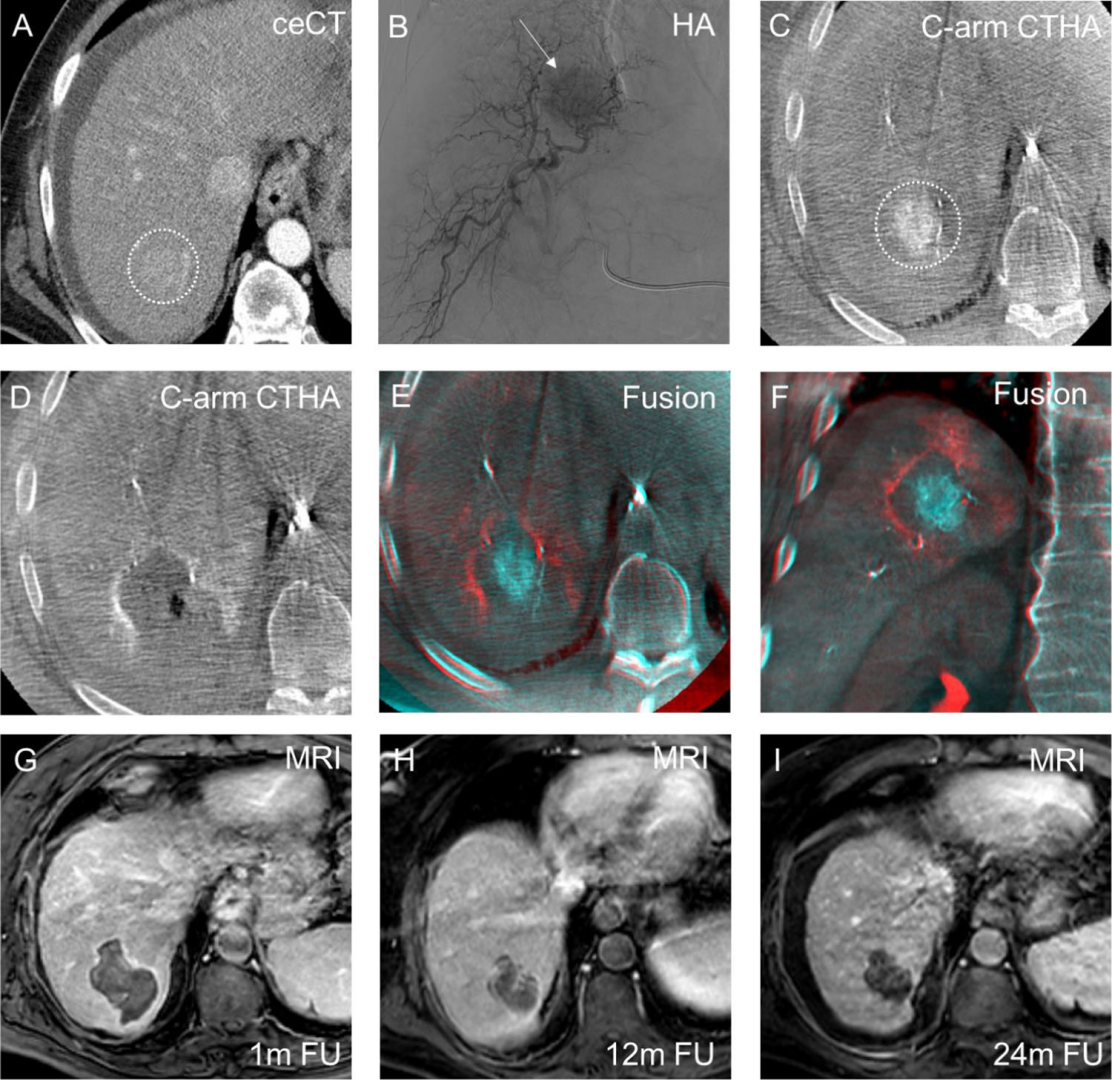
Example of a HepACAGA procedure for a HCC with a tumor size of 36 mm located in segment VII/VIII. **A** The lesion is moderately visible on contrast-enhanced CT (ceCT) during the arterial phase (white circle) 1 month prior to the procedure **B** The lesion is visualized with hepatic arteriography (HA) (white arrow) during the procedure **C** Intraprocedural C-arm CT hepatic arteriography (C-arm CTHA) provides adequate visualization of the target lesion (white circle) **D** The ablation zone after 15 min ablation at 150W is depicted with C-arm-CTHA immediately after ablation **E,F** Fusion of pre- and post-ablation C-arm CTHAs using XperGuide software in the axial and coronal planes confirms adequate ablation margins **G,H,I** Follow-up (FU) MRI (T1 with gadolinium) at 1 month, 12 months, and 24 months demonstrates no local tumor progression and a progressively shrinking ablation zone

All HepACAGA procedures were conducted with MWA using the Emprint HP microwave generator (Medtronic, Dublin, Ireland). Immediately following ablation, C-arm CTHA was repeated to visualize the ablation zone (similar to pre-ablation C-arm CTHA). Ablation margins were assessed by automatic fusion of pre- and post-ablation C-arm CTs with rigid registration (XperGuide). The aim was a minimal ablation margin of 5 mm. Overlapping ablations were performed if necessary using the next steps: 1. after initial ablation, a second needle trajectory was planned with XperGuide; 2. the needle was removed under tract ablation; 3. The needle was re-inserted according to the new trajectory. If margins were insufficient, additional ablation was conducted, with needle repositioning if needed. The procedure concluded with tract ablation, catheter removal, and puncture site closure. Figure 1 illustrates a HepACAGA procedure for a 36 mm tumor.

### Follow-up Imaging

Follow-up imaging consisted of an initial CT or MRI 1 month post-HepACAGA to assess tumor recurrence or residual tumor tissue at the site of the ablation zone. Subsequent imaging (CT, MRI, or ^18^F-FDG PET/CT) was performed every 3–6 months for disease evaluation.

### Tumor Size Stratification

To assess the impact of tumor size on outcomes of HepACAGA, the cohort was stratified into three subgroups based on tumor size: lesions ≤ 2 cm, 2–3 cm (i.e., > 2 cm and ≤ 3 cm), and 3–5 cm (i.e., > 3 cm and ≤ 5 cm). Tumor size was determined using a one-dimensional linear measurement of the largest diameter on intraprocedural C-arm CT. Patients were categorized into a subgroup based on their largest tumor, regardless of smaller additional tumors. Similarly, procedures were categorized based on the largest tumor treated during the session.

## Study Objectives

### Local Tumor Progression

The primary objective was to compare local tumor progression-free survival (LTPFS), including 1-year LTPFS rates, across the tumor size subgroups. Any indication of residual or recurrent tumor tissue at the site of the ablation zone, based on the radiology reports, was considered LTP. LTPFS was defined as the period of time from the date of ablation until observation of local progression of the tumor. Non-recurrent tumors were censored at the date of last follow-up assessment.

LTP rates were presented as the percentage of recurrent tumors at the ablation site during follow-up, and were compared between subgroups. Primary LTP was defined as the proportion of lesions that recurred after the initial ablation, while secondary LTP referred to the number of tumors that still recurred following re-ablations. LTPFS and LTP were evaluated with a per-tumor analysis.

Overall survival (OS) was defined as the duration from the date of ablation until the event of death by any cause.

### Technical Success

Technical success was defined as intraprocedural detection of the target lesion(s) and its subsequent ablation with margins that were deemed adequate (aiming for ≥ 5 mm). Patient records and radiology reports were examined to assess technical success. Technical success was evaluated per procedure; if any tumor in a multi-lesion session was not successfully treated, the procedure was classified as technically unsuccessful.

### Complications

Complications were graded according to the Common Terminology Criteria for Adverse Events (CTCAE), v5 [19]. Perioperative mortality, defined as death occurring within 30 days after ablation, was also assessed.

### Statistical Analysis

Baseline characteristics for normally distributed continuous variables were summarized as means ± SD and compared using one-way ANOVA. Non-normally distributed variables were summarized as medians (IQR, interquartile range) and compared using Kruskal–Wallis tests. Categorical variables were presented as counts (percentages) and compared using Pearson’s Chi-Square (Χ^2^) tests. LTPFS and OS were estimated with Kaplan–Meier survival curves and compared using a log-rank test. Corresponding LTPFS hazard ratios (HR) were obtained with Cox proportional hazards regression analysis. LTP, technical success, and complication rates were compared across subgroups using Pearson Chi-Square (Χ^2^) tests. Statistical analyses were conducted using R version 4.3.2 (R Foundation, Vienna, Austria). *p*-values < 0.05 were considered statistically significant.

## Results

### Patient Selection

**Fig. 2:**
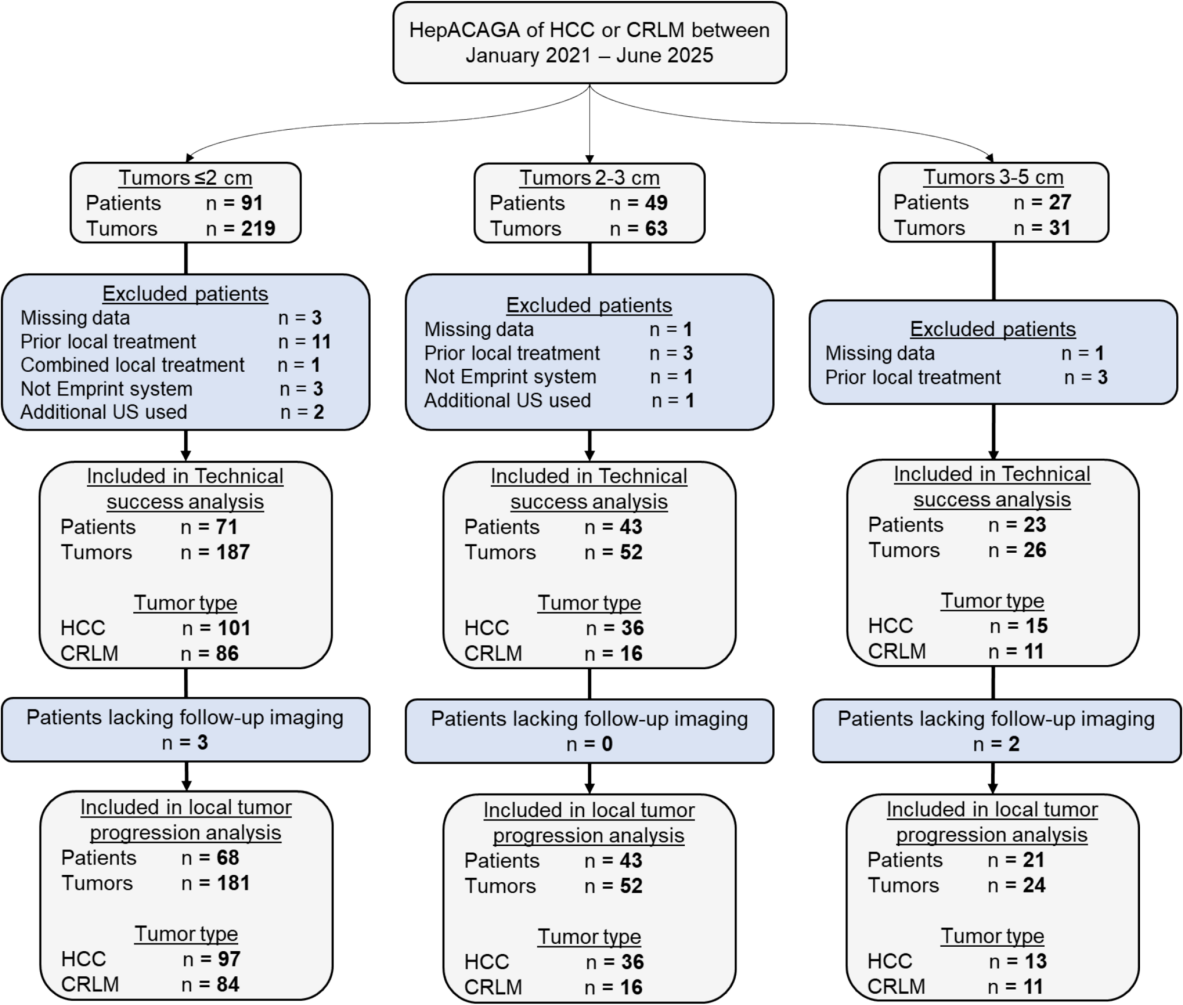
Flowchart demonstrating subgroup inclusion. Patients without follow-up imaging were only included in the technical success analysis

A total of 167 patients with 313 tumors (174 HCC, 139 CRLM) treated with HepACAGA were identified. After exclusions, 137 patients with 265 tumors (152 HCC, 113 CRLM) were included and stratified into three subgroups: 187 tumors ≤ 2 cm (101 HCC, 86 CRLM), 52 tumors 2–3 cm (36 HCC, 16 CRLM), and 26 tumors 3–5 cm (15 HCC, 11 CRLM) (Fig. 2).

Table 1 summarizes baseline characteristics. The overall median intraprocedural tumor size was 16 mm (IQR 11–22): 13 mm (IQR 10–16) for tumors ≤ 2 cm, 24 mm (IQR 22–26) for tumors 2–3 cm, and 35 mm (IQR 32–37) for tumors 3–5 cm.

**Table 1:** Baseline characteristics per subgroup

	All	≤ 2 cm	2–3 cm	3–5 cm	*p*-value
*Patient-related characteristics*	*n* = 137	*n* = 71	*n* = 43	*n* = 23	
Sex, *n* (%)					0.484†
Male	106 (77)	52 (73)	35 (81)	19 (83)	
Female	31 (23)	19 (27)	8 (19)	4 (17)	
Age (years), median (IQR)	68 (61–75)	67 (60–76)	67 (62–74)	67 (61–76)	0.909*
BMI (kg/m^2^), mean ± SD	27 ± 4	27 ± 5	28 ± 4	28 ± 5	0.612*
ASA score, *n* (%)					0.616†
1	7 (5)	3 (4)	3 (7)	1 (4)	
2	29 (21)	19 (27)	5 (12)	5 (22)	
3	85 (62)	43 (61)	28 (65)	14 (61)	
4	13 (9)	5 (7)	5 (12)	3 (13)	
NR	3 (2)	1 (1)	2 (5)	-	
Number of intrahepatic tumors at time of ablation, *n* (%)					0.715†
1	76 (55)	41 (58)	22 (51)	13 (57)	
2–3	49 (36)	26 (37)	16 (37)	7 (30)	
≥ 4	12 (9)	4 (6)	5 (12)	3 (13)	
*Tumor-related characteristics*	*n* = 265	*n* = 187	*n* = 52	*n* = 26	
Tumor type, *n* (%)					0.146†
HCC	152 (57)	101 (54)	36 (69)	15 (58)	
CRLM	113 (43)	86 (46)	16 (31)	11 (42)	
Intraprocedural tumor size (mm), median (IQR)	16 (11–22)	13 (10–16)	24 (22–26)	35 (32–37)	< 0.001*
Baseline imaging (MRI/CT) tumor size (mm), median (IQR)	13 (10–20)	11 (9–14)	22 (21–25)	31 (28–34)	< 0.001*
*Procedural characteristics*	*n* = 173	*n* = 102	*n* = 47	*n* = 24	
Power (Watt), mean ± SD	124 ± 27	118 ± 27	132 ± 24	150 ± 2	< 0.001*
Ablation duration (min), median (IQR)	6 (4–10)	5 (3–8)	9 (5–10)	11 (10–15)	< 0.001*
Antenna repositions, *n* (%)					0.737†
0	129 (75)	75 (74)	37 (79)	17 (71)	
1	41 (24)	25 (25)	10 (21)	6 (25)	
2	3 (2)	2 (2)	-	1 (4)	
Antenna repositioning to create larger overlapping ablation zones, n (%)	29 (17)	8 (8)	8 (17)	13 (54)	< 0.001*

ASA = American society of Anesthesiologists physical status, BMI = body mass index in kg/m^2^, CRLM = colorectal liver metastases, HCC = hepatocellular carcinoma, IQR = interquartile range, NR = not reported, SD = standard deviation. *Kruskal–Wallis test or one-way analysis of variance (ANOVA). †Chi-Square (χ^2^) test

### Follow-up Analysis

Five patients with 8 tumors lacked follow-up imaging and were only included in the technical success analysis. As a result, a total of 132 patients with a combined 257 tumors (146 HCC, 111 CRLM) were included in the follow-up analysis: 181 tumors ≤ 2 cm (97 HCC, 84 CRLM), 52 tumors 2–3 cm (36 HCC, 16 CRLM), and 24 tumors 3–5 cm (13 HCC, 11 CRLM) (Table 2). Median follow-up was 9 months (IQR 4–20) overall and similar across subgroups: 9 months (IQR 3–21) for tumors ≤ 2 cm, 10 months (IQR 4–17) for tumors 2–3 cm, and 9 months (IQR 4–17) for tumors 3–5 cm (*p* = 0.765). Median follow-up was 11 months for HCC and 9 months for CRLM.

**Table 2:** Follow-up related outcomes

	All	≤ 2 cm	2–3 cm	3–5 cm	*p*-value
*Follow-up per tumor*	*n* = 257	*n* = 181	*n* = 52	*n* = 24	
Follow-up (months), median (IQR)	9 (4–20)	9 (3–21)	10 (4–17)	9 (4–17)	0.765*
Imaging modality of follow-up, *n* (%)					0.925†
MRI only	150 (58)	109 (60)	28 (54)	13 (54)	
CT only	15 (6)	11 (6)	2 (4)	2 (8)	
MRI and CT	76 (30)	50 (28)	19 (37)	7 (29)	
MRI and ^18F^FDG-PET	9 (4)	7 (4)	1 (2)	1 (4)	
MRI, CT and ^18F^FDG-PET	7 (3)	4 (2)	2 (4)	1 (4)	
Primary LTP, *n* (%)	13 (5)	8 (4)	3 (6)	2 (8)	0.689†
Secondary LTP, *n* (%)	9 (4)	6 (3)	2 (4)	1 (4)	0.966†
LTP HCC	*n* = 146	*n* = 97	*n* = 36	*n* = 13	
LTP HCC, *n* (%)	4 (3)	1 (1)	2 (6)	1 (8)	0.189†
LTP CRLM	*n* = 111	*n* = 84	*n* = 16	*n* = 11	
LTP CRLM, *n* (%)	9 (8)	7 (8)	1 (6)	1 (9)	0.954†
*Follow-up per patient*	*n* = 132	*n* = 68	*n* = 43	*n* = 21	
New liver tumors (not at site of ablation zone), *n* (%)	58 (44)	29 (43)	19 (44)	10 (48)	0.922†
New or progressive extrahepatic disease, *n* (%)	28 (21)	15 (22)	6 (14)	7 (33)	0.199†

HCC = hepatocellular carcinoma, IQR = interquartile range, LTP = local tumor progression. *Kruskal–Wallis test. †Chi-Square (χ^2^) test. Note that, compared to the baseline table, there are fewer patients and tumors due to a lack of follow-up

**Fig. 3:**
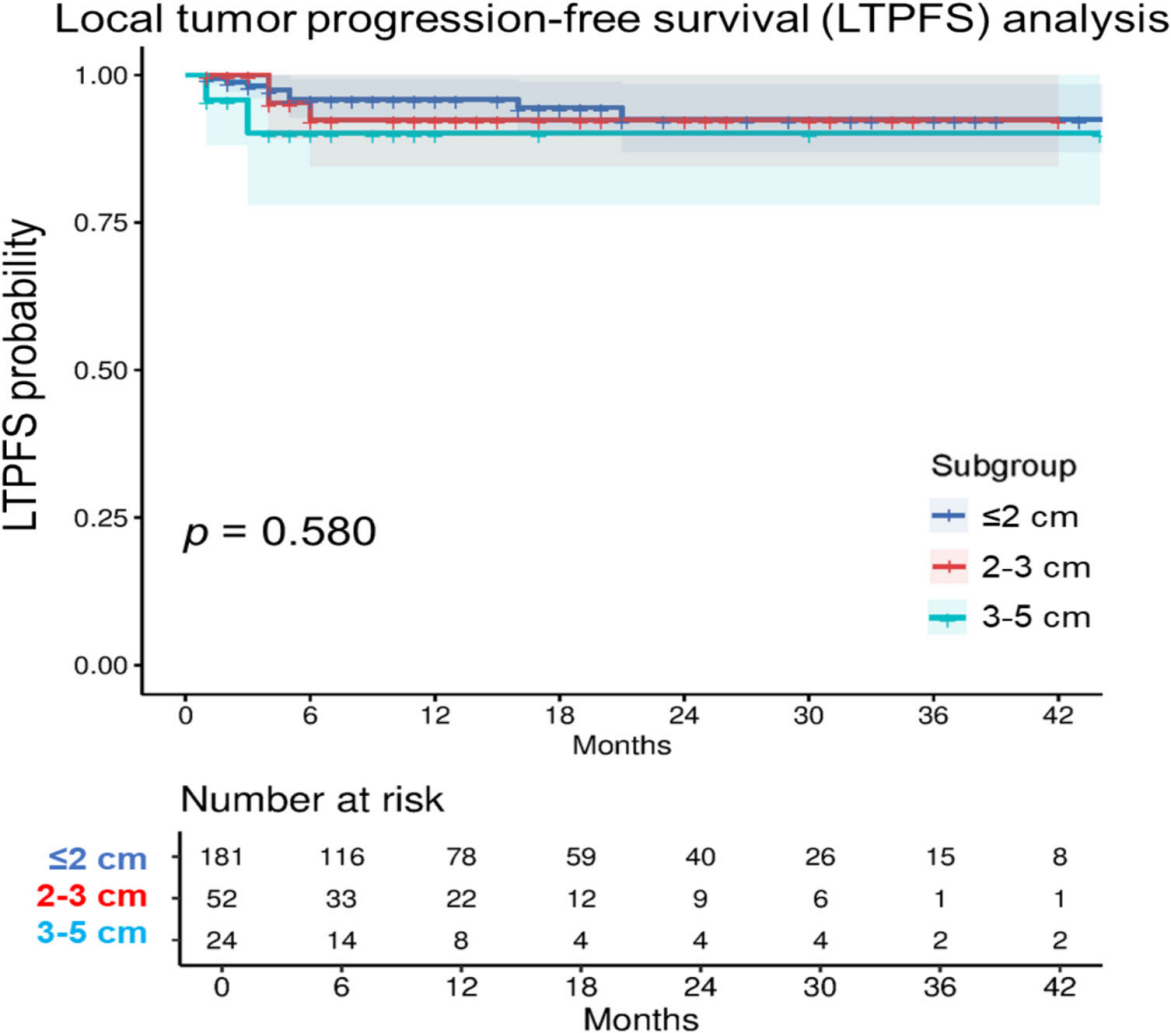
Kaplan–Meier curves of per-tumor analysis of local tumor progression-free survival (LTPFS) with 95% confidence intervals. Number at risk refer to the number of tumors present at each time point. A log-rank test was used for subgroup comparison

The 1-year LTPFS rate was most favorable for tumors ≤ 2 cm (96%; 95% CI: 93–99), followed by tumors 2–3 cm (93%; 95% CI: 85–100), and tumors 3–5 cm (90%; 95% CI: 78-100). Kaplan–Meier curves showed no significant difference in per-tumor LTPFS between tumor size subgroups (*p* = 0.580) (HR 2–3 cm vs. ≤ 2 cm: 1.33 [95% CI: 0.35–5.02], *p* = 0.676; HR 3–5 cm vs. ≤ 2 cm: 2.11 [95% CI: 0.45–9.96], *p* = 0.345) (Fig. 3). Median LTPFS was not reached. Supplementary Materials present LTPFS by tumor type (Figure S1, Table S1).

Overall, LTP occurred in 13/257 tumors (5%). Of the 13 tumors with LTP, adequate ablation margins were achieved in 1 case; recurrence in this patient was most likely attributable to tumor seeding caused by deep needle placement. In the remaining 12 cases, ablation margins were inadequate. In 4 out of these 12 cases (33%), achieving larger ablation margins was not safely possible due to the tumors being close to critical structures (e.g., gallbladder). The primary LTP rates were 4% for tumors ≤ 2 cm (8/181), 6% for tumors 2–3 cm (3/52), and 8% for tumors 3–5 cm (2/24) (*p* = 0.689). Four recurrent tumors were successfully re-ablated; the resulting secondary LTP rates were 3% for tumors ≤ 2 cm (6/181), and 4% for both tumors 2–3 cm (2/52) and tumors 3–5 cm (1/24) (*p* = 0.966). LTP subgroup analyses for HCC and CRLM are shown in Table 2.

There was no perioperative or procedure-related mortality. Median OS was not reached for HCC or CRLM. OS Kaplan–Meier curves are presented for each tumor type in the Supplementary Materials (Figure S2, Table S1).

New liver tumors outside the targeted area were detected in 58/132 patients (44%): 34/78 (44%) with HCC and 24/54 (44%) with CRLM. New or progressive extrahepatic disease was observed in 28/132 patients (21%): 9/78 (12%) with HCC and 19/54 (35%) with CRLM. There were no statistically significant differences in new intra- or extrahepatic disease among subgroups.

### Procedure-Related Outcomes

**Fig. 4:**
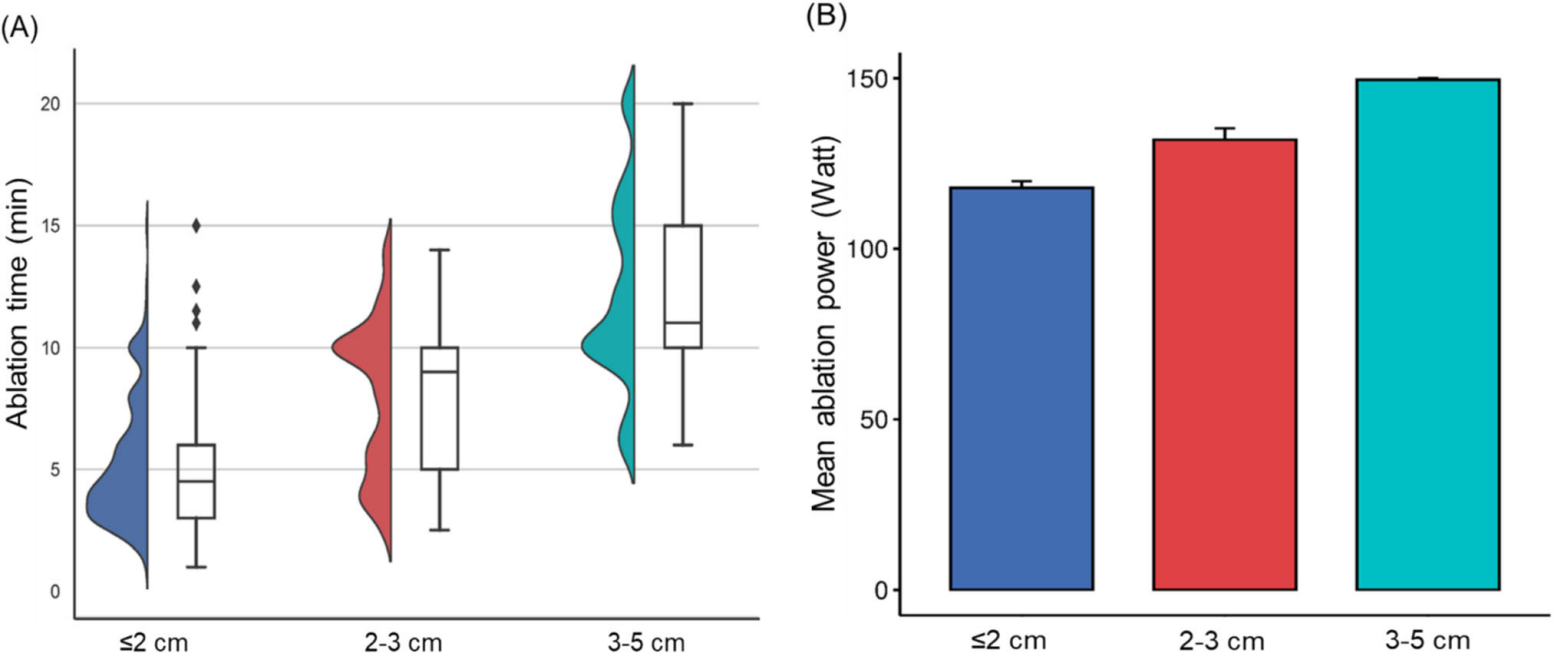
**A** Boxplot and violin plot of ablation duration (min) for each subgroup. **B** Bar chart of mean ablation power in Watt for each subgroup

Ablation duration and power increased with tumor size (Fig. 4). Median ablation duration was 5 min (IQR 3–8) for tumors ≤ 2 cm, 9 min (IQR 5–10) for tumors 2–3 cm, and 11 min (IQR 10–15) for tumors 3–5 cm (*p* < 0.001) (Table 1). Mean power was 118W (± 27W) for tumors ≤ 2 cm, 132W (± 24W) for tumors 2–3 cm, and 150W (± 2W) for tumors 3–5 cm (*p* < 0.001).

Technical success was achieved in 172/173 procedures (99%) (Table 3). One failure in the 3–5 cm group occurred due to inability to separate the stomach from the liver, despite hydrodissection and pneumodissection.

**Table 3:** Procedure-related outcomes

	All	≤ 2 cm	2–3 cm	3–5 cm	*p*-value
*Technical success*	*n* = 173	*n* = 102	*n* = 47	*n* = 24	
Technical success, *n* (%)	172 (99)	102 (100)	47 (100)	23 (96)	0.044†
*Complications*	*n* = 172	*n* = 102	*n* = 47	*n* = 23	
Complicated procedures, *n* (%)	10 (6)	4 (4)	3 (6)	3 (13)	0.236†
Complications (CTCAE v5), *n* (%)					
CTCAE grade 1	1 (1)	–	–	1 (4)	
CTCAE grade 2	7 (4)	4 (4)	2 (4)	1 (4)	
CTCAE grade 3	2 (1)	–	1 (2)	1 (4)	
Type of complication, *n* (%)					
Pneumothorax (no intervention)	1 (1)	–	–	1 (4)	
Pleural effusion with collapse of right lower lobe	1 (1)	–	–	1 (4)	
Bleeding requiring embolization	6 (3)	3 (3)	2 (4)	1 (4)	
Postprocedural infection (requiring drainage of ablation zone)	1 (1)	–	1 (2)	–	
Arterial dissection	1 (1)	1 (1)	–	–	

CTCAE v5 = Common Terminology Criteria for Adverse Events version 5.0. †Chi-Square (χ^2^) test

Complications occurred overall in 10/172 procedures (6%) (Table 3). The complication rate was lowest for procedures involving tumors ≤ 2 cm (4/102, 4%), followed by tumors 2–3 cm (3/47, 6%), and tumors 3–5 cm (3/23, 13%) (*p* = 0.236). The most common complication was bleeding requiring embolization (6/172, 3%).

## Discussion

This cohort study demonstrated a low overall LTP rate of 5%, regardless of tumor size. One-year LTPFS after HepACAGA was favorable across all subgroups: 96% for tumors ≤ 2 cm, 93% for tumors 2–3 cm, and 90% for tumors 3–5 cm.

The results demonstrated low LTP rates and durable LTPFS across a broad range of tumor sizes, including tumors 3–5 cm. While treatment efficacy slightly decreased with increasing tumor size, outcomes remained acceptable: for tumors 3–5 cm, the primary LTP rate was 8%, the secondary LTP rate was 4%, and 1-year LTPFS was 90%. These findings support thermal ablation as a viable option for tumors 3–5 cm and can be used to make more informed decisions during multidisciplinary tumor board meetings. Instead of focusing on an absolute tumor diameter, we recommend offering thermal ablation to patients when adequate ablation margins can be achieved. This needs to be assessed on a case-by-case basis, and will depend on tumor location and available ablation techniques.

Another study comparing CRLM ablation outcomes for tumors ≤ 3 cm and 3–5 cm found a higher 1-year LTPFS for tumors ≤ 3 cm than for 3–5 cm tumors (93% vs. 75%, respectively) [20]. However, subanalysis showed significant LTPFS improvement for 3–5 cm tumors over time, attributed to technical advancements. A 10-year analysis of the same cohort demonstrated remarkable improvements in percutaneous thermal ablation efficacy: 2-year LTPFS increased from 37% in 2010–2013 to 86% in 2018–2021. This improvement was largely attributed to technological advancements, including more accurate needle placement techniques (e.g., CT hepatic arteriography), better ablation confirmation methods (e.g., fusion software), and higher energy delivery systems (e.g., high-power MWA) [21]. These findings are in agreement with the results of this study.

The overall complication rate was low (6%), with CTCAE grades ranging from 1 to 3 (no complications exceeding grade 3) and without procedure-related or perioperative mortality. Tumors 3–5 cm had a higher complication rate (13%), consisting of one CTCAE grade 1 (asymptomatic pneumothorax), one CTCAE grade 2 (bleeding requiring embolization), and one CTCAE grade 3 (pleural effusion with collapse of right lower lobe). This may be attributable to longer ablation durations and higher power settings for tumors 3–5 cm, increasing the risk of collateral damage. However, the small sample size in the 3–5 cm subgroup limits the ability to draw reliable conclusions.

The COLLISION trial recently demonstrated the non-inferiority of thermal ablation compared to surgical resection for CRLM ≤ 3 cm (each group: 148 patients), showing similar per-tumor LTP rates (surgery: 8% vs. ablation: 7%) and no statistically significant differences in LTPFS, while ablation had a significantly better safety profile (complication rate: surgery 46% vs. ablation 19%) [22]. For tumors 3–5 cm, surgical resection has traditionally been the preferred treatment with superior outcomes compared to thermal ablation [23–25]. However, the outcomes of thermal ablation have improved significantly with recent technological advances such as the HepACAGA technique [11–15]. In this study, the primary LTP rate for intermediate-sized tumors (3–5 cm) was 8%, equivalent to the surgical resection group in the COLLISION trial for smaller tumors. An advantage of thermal ablation is its suitability for repeat ablations in cases of LTP, compared to surgery or stereotactic body radiotherapy (SBRT), where repeatability is often more limited [26].

SBRT is a valuable alternative to thermal ablation. At this moment, it is mostly applied when thermal ablation is contraindicated due to an unfavorable tumor location [27]. In a retrospective study, thermal ablation demonstrated superior OS and LTPFS compared to SBRT in CRLM with a slightly higher risk of serious adverse events [28]. Other retrospective studies comparing SBRT with RFA (no MWA) found that SBRT provided better local control for lesions ≥ 2 cm (HCC and metastases), suggesting its potential efficacy for larger tumors [29, 30]. It remains unclear whether thermal ablation or SBRT is the preferred option for intermediate-sized lesions (3–5 cm). The ongoing COLLISION-XL randomized trial, comparing MWA and SBRT for unresectable CRLM (3–5 cm), aims to provide clarity [31].

This study has several limitations. The primary limitation is the small sample size of tumors 3–5 cm, which may reduce statistical power and generalizability of the findings. Selection bias has likely played a role since not all patients with 3–5 cm tumors at our institute were referred for ablation in the study period (primarily unsuitable candidates for surgery). Another limitation is the inclusion of both HCC and CRLM within the same cohort. In patients with CRLM, primary tumor location (right- vs. left-sided colon) may influence clinical outcomes. Although efficacy outcomes were reported separately for HCC and CRLM, the limited subgroup sizes constrain the ability to draw definitive conclusions for each tumor type individually, or to assess the impact of primary tumor sidedness in CRLM. Moreover, patients and procedures were categorized into subgroups based on the size of the largest tumor at the time of ablation, without accounting for additional smaller tumors. This may have introduced confounding. Another limitation is the short median follow-up time (9 months overall, IQR: 4–20), limiting the ability to detect long-term LTP, although most LTP typically occurs within the first three to nine months post-ablation [32].

Future studies, preferably disease-specific and with larger cohorts and longer follow-up, are warranted to validate these findings and guide evidence-based adjustments to current treatment guidelines.

## Conclusion

This study demonstrated that HepACAGA is an effective thermal ablation technique for HCC and CRLM, providing durable LTPFS and low LTP rates for tumors up to 5 cm in this cohort. These findings support considering thermal ablation as a potential option also for tumors with a diameter between 3 and 5 cm, particularly when adequate ablation margins can be achieved and sufficient local expertise is available.

## Supplementary Information

Below is the link to the electronic supplementary material.

Supplementary file1 (DOCX 358 KB)
