# Supplementary material for: Impact of Tumor Size on Outcomes of Hepatic Arteriography and C-Arm CT-Guided Ablation (HepACAGA): > 3 cm Is No Absolute Contraindication
Source: Cardiovasc Intervent Radiol. 2025 Aug 26;49(1):59–69. doi: 10.1007/s00270-025-04167-8 (PMC12748118; doi:10.1007/s00270-025-04167-8)
Supplement: Supplementary file 1 — Supplementary file1 (DOCX 358 KB) [file 270_2025_4167_MOESM1_ESM.docx]

Electronic Supplementary Material


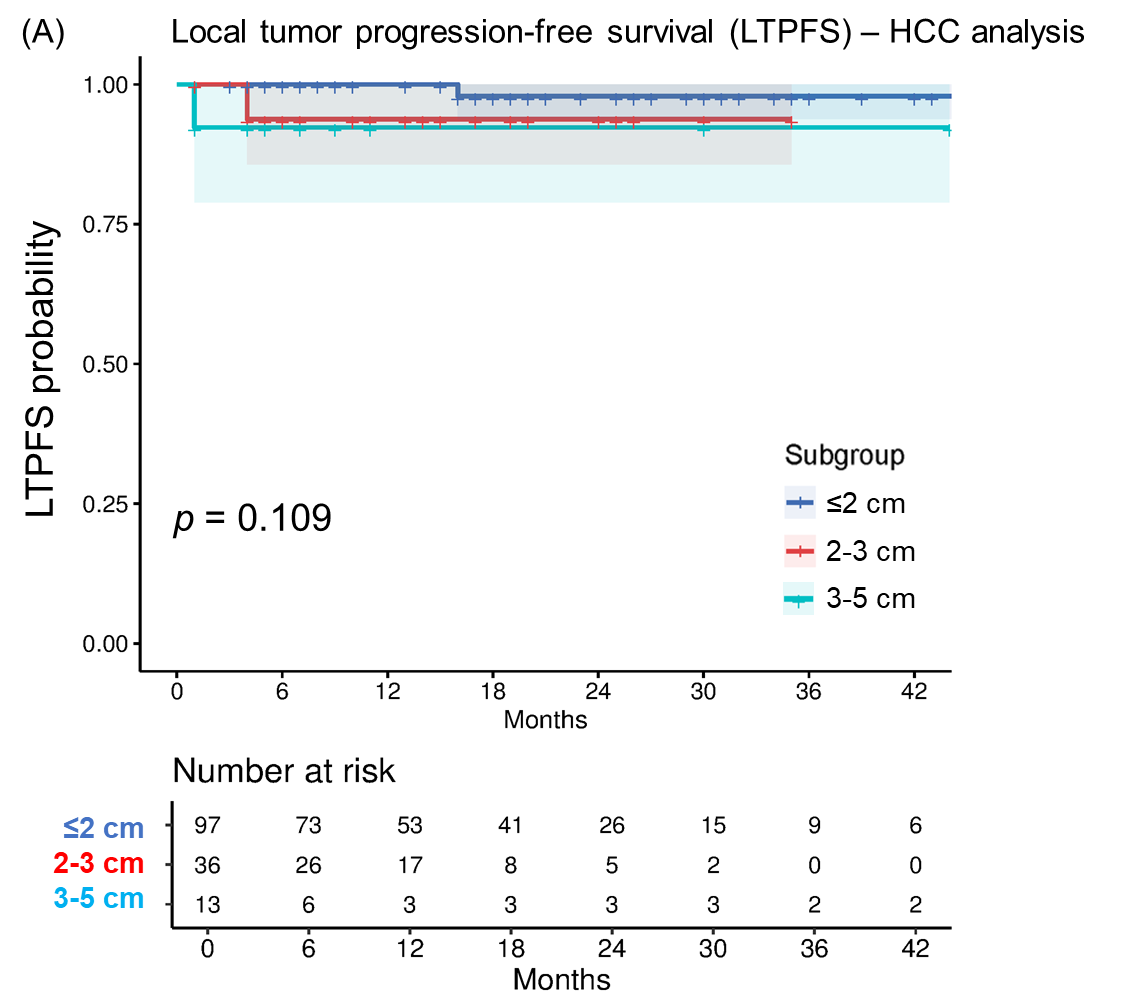


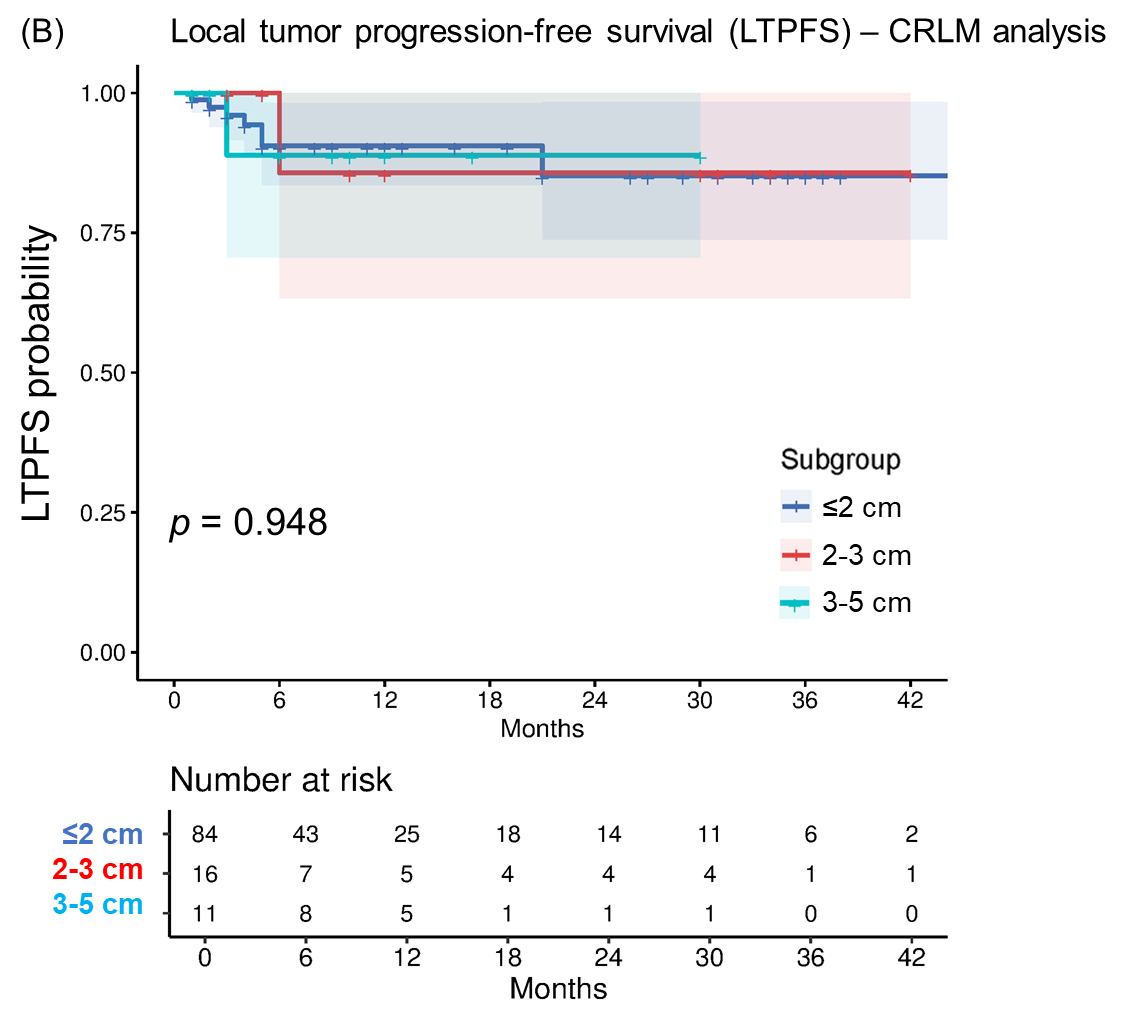


Figure S1. Kaplan–Meier survival curves illustrating the local tumor progression-free survival (LTPFS) with 95% CI for both tumor types separately. Log-rank tests were used for comparison. The number at risk corresponds to either the number of HCC or CRLM present at each time point. (A) represents the HCC analysis; (B) demonstrates the CRLM analysis.


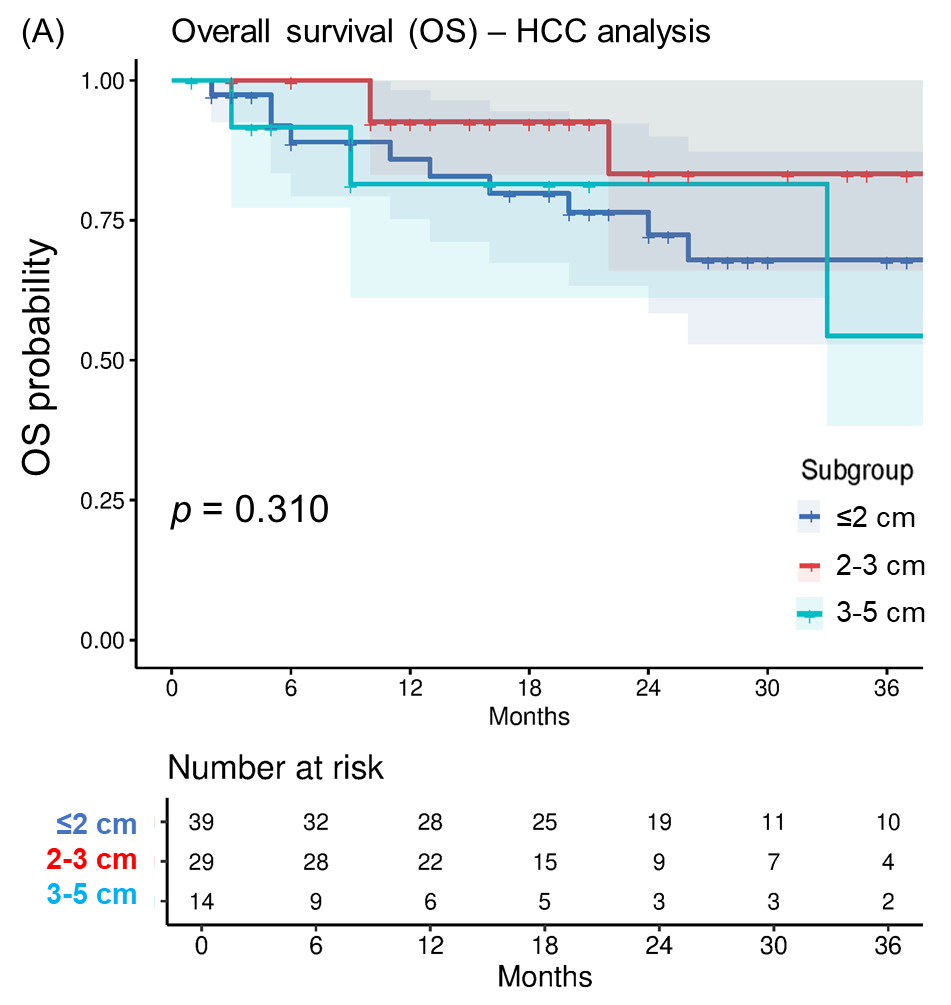


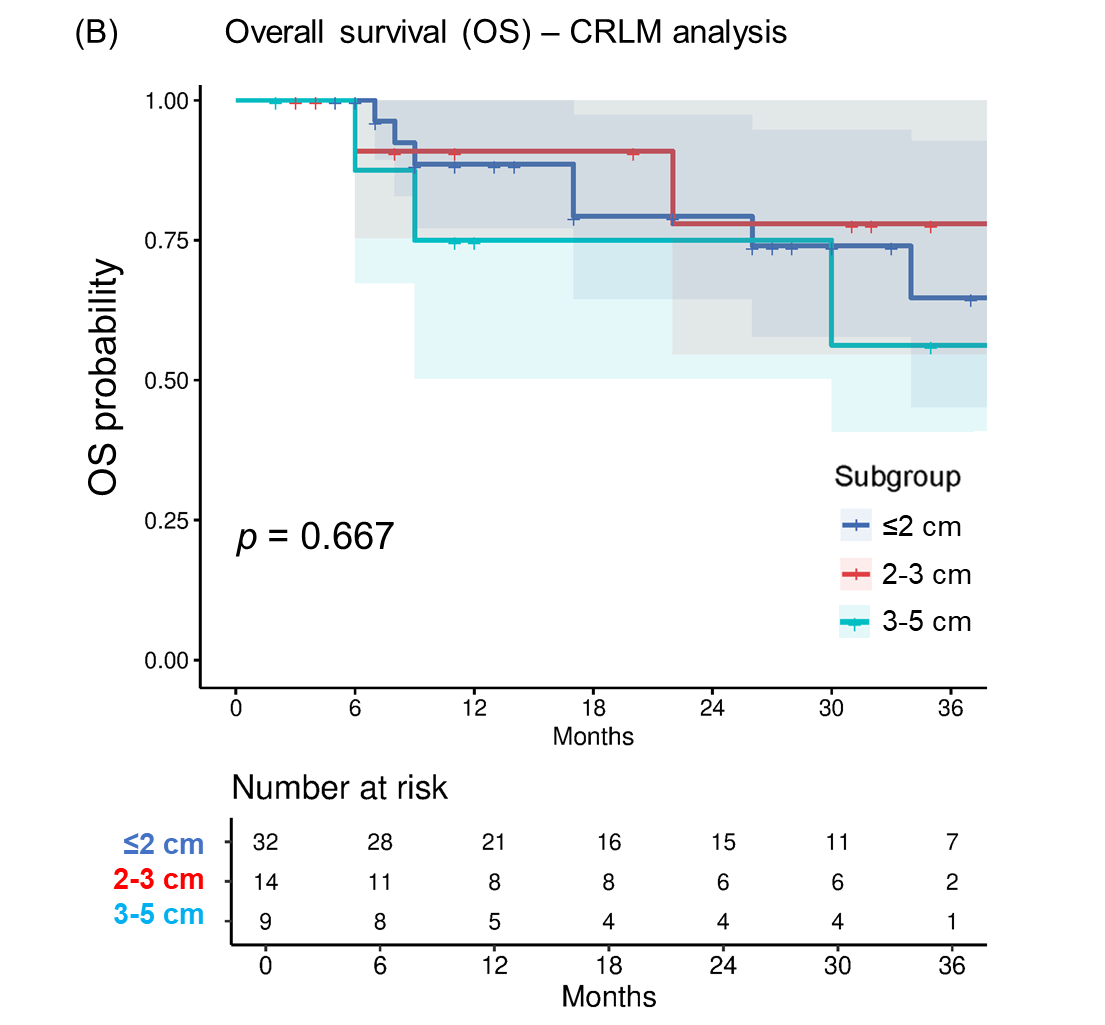


Figure S2. Kaplan–Meier survival curves illustrating the overall survival (OS) with 95% CI for both tumor types separately. Log-rank tests were used for comparison. The number at risk corresponds to either the number of HCC or CRLM patients present at each time point. (A) represents the HCC analysis; (B) demonstrates the CRLM analysis.

Table S1. 1-year LTPFS and 1-year OS rates per tumor type

|  | ≤ 2 cm | 2-3 cm | 3-5 cm |
| --- | --- | --- | --- |
| *1-year LTPFS (95% CI) – HCC* | 100% | 94% (86-100) | 92% (79-100) |
| *1-year LTPFS (95% CI) – CRLM* | 91% (84-98) | 86% (63-100) | 89% (71-100) |
| *1-year OS (95% CI) – HCC* | 86% (75-98) | 93% (83-100) | 81% (61-100) |
| *1-year OS (95% CI) – CRLM* | 89% (77-100) | 91% (75-100) | 75% (50-100) |

95% CI = 95% confidence interval, CRLM = colorectal liver metastases, HCC = hepatocellular carcinoma, LTPFS = local tumor progression-free survival, OS = overall survival.
